# RelabotulinumtoxinA, a Ready-to-Use Formulation Neuromodulator Manufactured with PEARL™ Technology to Maintain High Biological and Specific Activity

**DOI:** 10.3390/toxins17100501

**Published:** 2025-10-09

**Authors:** Ulf Ståhl, Emilia Lekholm, Emil Hamnevik, Robert Fredriksson, Sachin M. Shridharani, Keywan Taghetchian, Joel L. Cohen, Mark S. Nestor, Åsa Liljegren Sundberg

**Affiliations:** 1 Galderma, 752 28 Uppsala, Sweden; 2 Department of Pharmaceutical Biosciences, Uppsala University, 751 24 Uppsala, Sweden; 3 School of Medicine, Washington University, Saint Louis, MO 63110, USA; 4 LUXURGERY, New York, NY 10022, USA; 5 Smoothline, 80331 Munich, Germany; 6 About Skin Dermatology, Greenwood Village, CO 80111, USA; 7 Department of Dermatology, University of California, Irvine, CA 92697, USA; 8 Center for Clinical and Cosmetic Research, Aventura, FL 33180, USA; 9 Department of Dermatology and Cutaneous Surgery, University of Miami Miller School of Medicine, Miami, FL 33125, USA; 10 Department of Surgery, University of Miami Miller School of Medicine, Miami, FL 33136, USA

**Keywords:** botulinum toxin type A, enzyme activity, onabotulinumtoxinA, biological activity, ready-to-use, relabotulinumtoxinA, specific activity

## Abstract

Most botulinum toxin A (BoNT-A) products for esthetic use require reconstitution before administration. Ready-to-use relabotulinumtoxinA is a liquid manufactured using Precipitation-free Extraction and Activity-preserving, Refined Liquid (PEARL™) Technology from a proprietary *C. botulinum* type A1 strain. We examined the in vitro characteristics of relabotulinumtoxinA. The specific BoNT-A1 potency remained consistent throughout drug substance manufacturing (1.9 × 10^8^–2.2 × 10^8^ LD_50_ mouse potency units/mg of BoNT-A1, four fractions sampled). Using glabellar line (GL) on-label doses, relabotulinumtoxinA liquid product was compared with powder onabotulinumtoxinA using the following: BoNT-A1 amount based on ELISA; specific enzyme activity based on SNAP-25 cleavage by a fluorescence resonance energy transfer-based assay (BoTest^®^); biological activity (binding, internalization, and SNAP-25 cleavage over time) using a cell-based assay. RelabotulinumtoxinA contained more BoNT-A1 per on-label GL dose (0.27 ng) than onabotulinumtoxinA (0.18 ng), had higher enzyme activity (53 vs. 29 BoTest^®^ units) per GL dose, and had higher specific activity per pg BoNT-A, with onabotulinumtoxinA displaying 81% of the specific activity of relabotulinumtoxinA. In vitro, relabotulinumtoxinA demonstrated higher biological activity and earlier onset of SNAP-25-cleavage than onabotulinumtoxinA. PEARL^TM^ Technology thus produces high-quality BoNT-A1 with high specific enzyme and biological activities, which may explain the clinical performance of relabotulinumtoxinA in Phase 3 clinical trials examining treatment of GLs and/or LCLs.

## 1. Introduction

Derived from *Clostridium botulinum* bacteria, botulinum neurotoxin serotype A (BoNT-A) products have a wide range of uses owing to their ability to induce temporary muscle paralysis [1,2,3,4,5]. BoNT-A causes its paralytic effect by inhibiting neuronal signaling at neuromuscular junctions [4,6]. More specifically, BoNT-A molecules block the release of the neurotransmitter acetylcholine through a series of actions that start with binding of BoNT-A to nerve terminals, followed by internalization inside synaptic vesicles, translocation of the light chain of the BoNT-A molecule across the vesicle membrane and release into the cytosol, and, finally, cleavage of the soluble N-ethylmaleimide-sensitive factor attachment receptor (SNARE) protein, synaptosome-associated protein 25 substrate (SNAP-25), which inhibits acetylcholine release [2,4].

Among the eight subtypes of the BoNT-A serotype, BoNT-A subtype 1 (BoNT-A1) has shown particular potency, and several BoNT-A1 products have now been developed for use in therapeutic settings [2,4,7,8]. The first commercially available BoNT-A1 product, onabotulinumtoxinA (onaBoNT-A [Botox^®^/Vistabel^®^], Allergan Aesthetics, an AbbVie Company, Irvine, CA, USA), received US Food and Drug Administration approval in 1989 for the treatment of blepharospasm and strabismus [9] and in 2002 for esthetic use in improving the appearance of moderate-to-severe frown lines (also known as glabellar lines [GLs]) [10,11]. Since then, additional BoNT-A1 products have been approved for use in the US and EU, including abobotulinumtoxinA (aboBoNT-A [Dysport^®^/Azzalure^®^], Ipsen Ltd., Slough, UK/Galderma SA, Lausanne, Switzerland) in 1990 for spasticity, in 2009 for esthetic use, and then in 2021 as a new formulation (Alluzience, Ipsen Ltd., Slough, UK/Galderma SA, Lausanne, Switzerland) [12,13]; incobotulinumtoxinA (incoBoNT-A [Xeomin^®^/Bocouture^®^], Merz Pharmaceuticals Inc., Frankfurt, Germany) [14,15]; and, more recently, prabotulinumtoxinA (praBoNT-A [Jeuveau^®^/Nuceiva^®^], Evolus, Newport Beach, CA, US/Daewoong, Seoul, Republic of Korea) [16]—all derived from the original Hall strain and then each having been modified to some extent in the manufacturing process. The approved uses of BoNT-A1 products have expanded in a range of both therapeutic (e.g., cervical dystonia, upper and lower limb spasticity, incontinence, chronic migraine) and facial esthetic indications [9,11,12,13,14,15,16,17,18].

There are several current challenges to BoNT-A1 use, not least that the formulations and production of BoNT-A1 products differ across manufacturers [1,2,19,20]. As a result, different BoNT-A1 products have distinct pharmacological characteristics and vary in their clinical performance. Furthermore, BoNT-A1 products are not interchangeable in terms of dosing units [19]. For example, the on-label doses used for the treatment of GLs are 20 U onaBoNT-A and 50 U aboBoNT-A [11,12,13,17]. In terms of manufacturing, most BoNT-A1 products are produced using precipitation for initial purification and lyophilization or vacuum-drying to eliminate the water from the formulation to obtain a powder and stabilize the final product. The powder-based BoNT-A1 products require reconstitution before use [11,12,13,15,18], which can lead to several potential challenges, including more time being required to prepare the product for injection and issues with reconstitution (foaming, inaccuracy in saline volume used for reconstitution, consistency, and precision of dosing) [21,22,23,24,25].

**Figure 1:**
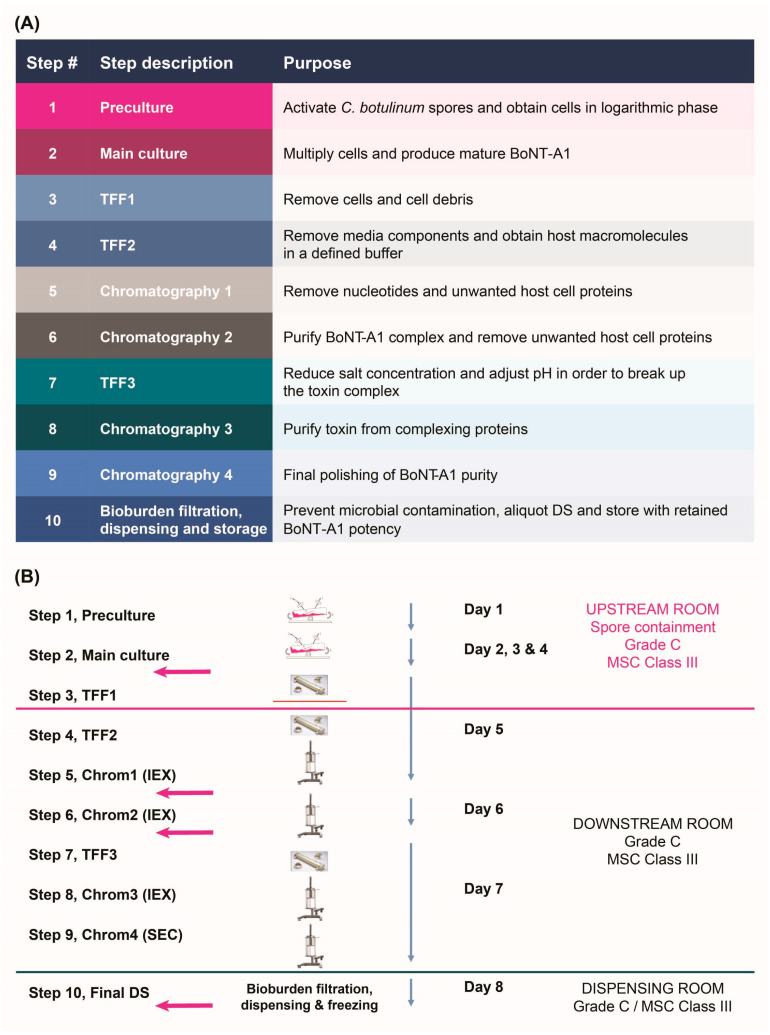
PEARL™ manufacturing process steps and sampling points. (**A**) Overview of process steps; (**B**) Timing of process steps. The pink arrows show timepoints of sampling for potency assessments during drug substance (DS) processing. BoNT-A1, botulinum toxin serotype A subtype 1; Chrom, chromatography; DS, drug substance; IEX, ion exchange chromatography; MSC, microbial safety cabinet; PEARL™, Precipitation-free Extraction and Activity-preserving, Refined Liquid; SEC, size exclusion chromatography; TFF, tangential flow filtration.

Following 30 years with few major innovations in the formulations of BoNT-A1 products [26], new formulations of BoNT-A1 are now being developed, with the aim of improving the performance and ease of use of currently available BoNT-A1 preparations. RelabotulinumtoxinA (relaBoNT-A [Relfydess™], Galderma, Uppsala, Sweden) is a recently developed novel-formulation, liquid, ready-to-use BoNT-A1 [27,28], which is approved in some markets for the treatment of adults aged over 18 years with moderate-to-severe GLs and lateral canthal lines (LCLs) [29,30]. RelaBoNT-A is produced using Precipitation-free Extraction and Activity-preserving, Refined Liquid (PEARL™) manufacturing technology, from a proprietary *Clostridium botulinum* type A1 strain [27,28]. With this process, the extraction and purification of BoNT-A1 is performed by using multiple tangential flow filtration and chromatography steps (see Section 5 and Figure 1). The technology allows the core botulinum toxin protein to be maintained in liquid form in its original conformation throughout the entire manufacturing process, which reduces any denaturation or unfolding that might occur during state changes, to preserve the core botulinum protein activity. The PEARL™ manufacturing process allows for a product that is highly pure, only containing 150 kDa core neurotoxin that is free of complexing proteins. In approved markets (the EU and Australia), each batch of relaBoNT-A is tested using a cell-based potency assay to ensure consistent high quality before commercial release. RelaBoNT-A treatment has previously shown significant improvements in the appearance of moderate-to-severe GLs and LCLs in recent randomized, double-blind, placebo-controlled, Phase 3 trials [31,32,33].

This article presents the in vitro characterization of ready-to-use relaBoNT-A, including the specific BoNT-A1 potency that is maintained throughout the relaBoNT-A drug substance manufacturing process, and the amount of BoNT-A core protein, specific enzyme (endoprotease) activity, and biological activity of the final drug product. The biological activity of relaBoNT-A was determined using neuronal cell-based assays of BoNT-A1 binding, internalization, and proteolytic cleavage of the SNARE protein, SNAP-25—key steps in the action of BoNT-A1 on target muscles [4]. In addition, the results of the relaBoNT-A assays were compared with similar assays for the powder BoNT-A1, onaBoNT-A, using on-label GL doses for each drug.

## 2. Results

### 2.1. BoNT-A1-Specific Potency Throughout PEARL^TM^ Drug Substance Processing

**Figure 2:**
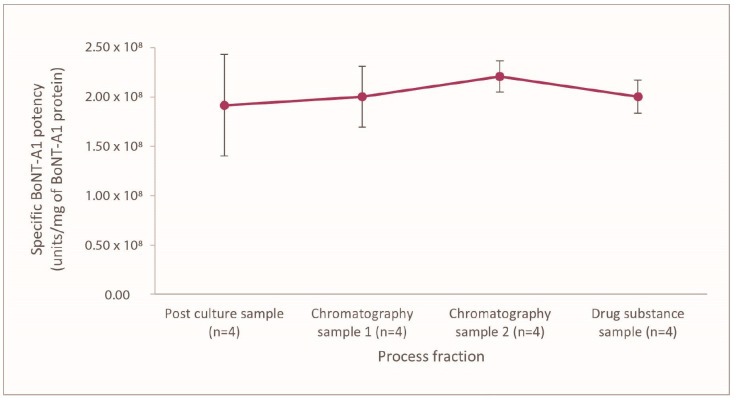
Specific BoNT-A1 activity for relaBoNT-A (per mg BoNT-A1), with mean values ± standard deviation, measured by mouse LD_50_ throughout the PEARL^TM^ manufacturing process for 4 different batches. BoNT-A1, botulinum toxin serotype A subtype 1; LD_50_, median lethal dose; relaBoNT-A, relabotulinumtoxinA.

The BoNT-A1-specific potency in the first collected fraction (initial post-culture fraction) during the PEARL^TM^ drug substance processing was ~2 × 10^8^ LD_50_ mouse units/mg. The BoNT-A1-specific potency for relaBoNT-A remained similar to the initial value of ~2 × 10^8^ LD_50_ mouse units/mg throughout the manufacturing process (1.9, 2.0, 2.2, and 2.0 LD_50_ units/mg in fractions 1, 2, 3, and 4) (Figure 2), thus showing that the drug substance (DS) manufacturing process allows for maintenance of the potency of the BoNT throughout the drug substance processing from initial bacterial culture to the final drug substance.

### 2.2. Characterization of Final relaBoNT-A Drug Product

#### BoNT-A Core Protein Amount, Enzyme Activity, and Biological Activity in the Final Drug Product

**Figure 3:**
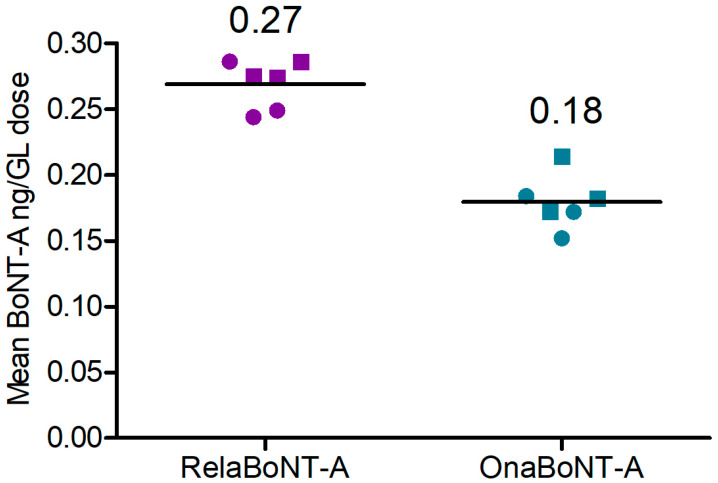
Amount of BoNT-A (ng) per GL dose for relaBoNT-A (50 U) and onaBoNT-A (20 U). Lines show the mean of three batches of each drug product, with each batch being measured with enzyme- linked immunosorbent assay (ELISA) at both Galderma (circles) and Uppsala University (squares). BoNT-A, botulinum toxin serotype A; GLs, glabellar lines; onaBoNT-A, onabotulinumtoxinA; relaBoNT-A, relabotulinumtoxinA; U, units.

The amount of BoNT-A core protein in the on-label GL dose (50 U) of final relaBoNT-A drug product was 0.27 ng, which was higher than in the on-label GL dose (20 U) of onaBoNT-A (0.18 ng), a between-product difference of 0.090 (99% CI: 0.06 to 0.12; effect size (ES) = 5.26, *p* < 0.01 (Figure 3, Table 1)).

**Figure 4:**
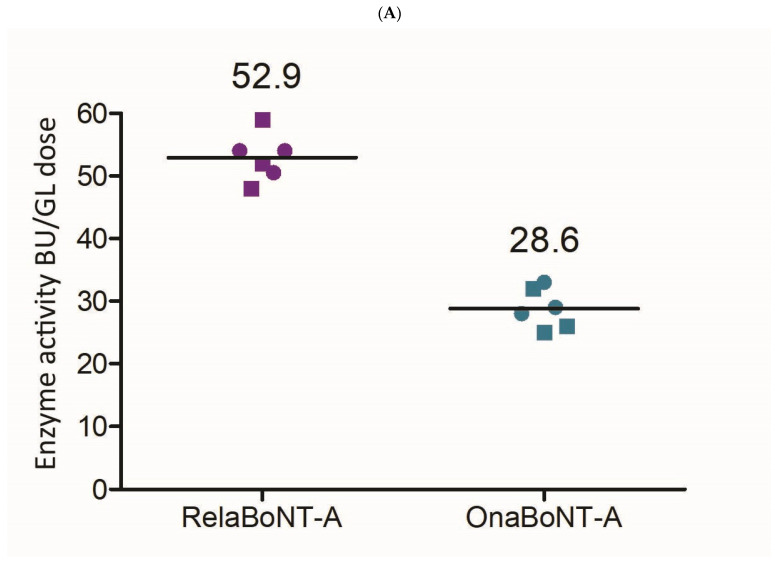

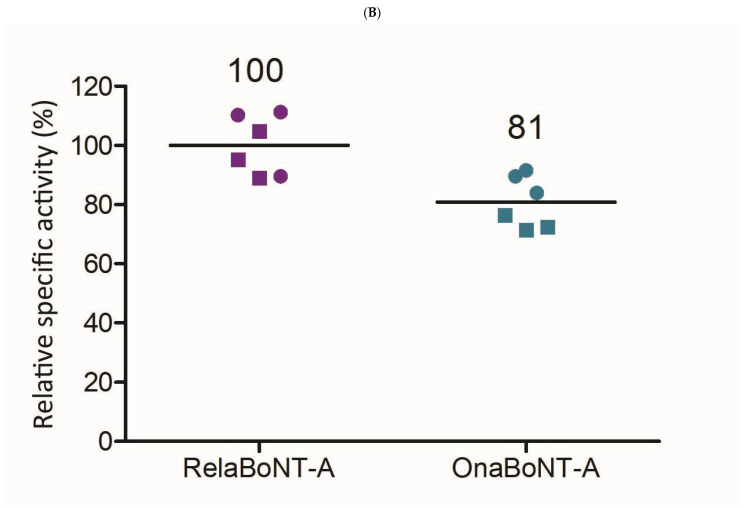
BoNT-A enzyme (endoprotease) activity. (**A**) Enzyme activity (BoTest^®^ units [BU]) per GL dose for relaBoNT-A (50 U) and onaBoNT-A (20 U). (**B**) Relative specific enzyme activity of relaBoNT-A (set to 100%) and onaBoNT-A. For (**A**,**B**), lines show the mean of three batches of each drug product, with each batch being measured at both Galderma (circles) and Uppsala University (squares). BoNT-A, botulinum toxin serotype A; BU, BoTest^®^ units; GLs, glabellar lines; onaBoNT-A, onabotulinumtoxinA; relaBoNT-A, relabotulinumtoxinA; U, units.

When assessing on-label GL doses, relaBoNT-A (50 U) showed higher enzyme activity than onaBoNT-A (20 U) (53 versus 29 BoTest^®^ units per GL dose, a between-product difference of 24.4; 99% CI: 17.3 to 31.4; ES = 6.52; *p* < 0.01) (Figure 4A, Table 1). In addition, the final relaBoNT-A drug product showed a higher specific enzyme activity (BoTest units/pg of core neurotoxin protein [ELISA]), with onaBoNT-A displaying 81% of the specific activity of relaBoNT-A (Figure 4B). The between-product difference in specific enzyme activity was 0.038 (99% CI: 0.009 to 0.066; ES = 2.50; *p* < 0.01 (Table 1)).

**Figure 5:**
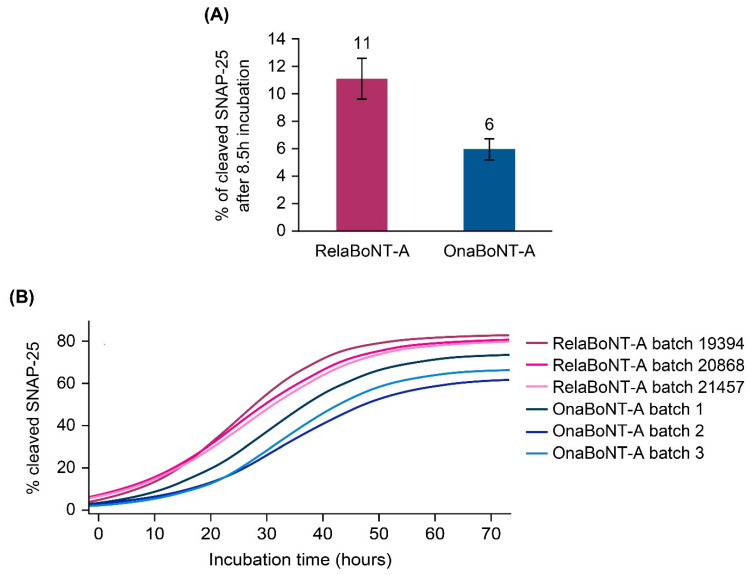
BoNT-A’s biological activity, measured in terms of percent of cleaved SNAP-25 in a motor neuron cell-based assay, following incubation with each BoNT-A drug product. BoNT-A doses corresponded to the glabellar line injection dose for each product (50 U relaBoNT-A and 20 U onaBoNT-A). (**A**) BoNT-A’s biological activity after 8.5 h of incubation with 50 U relaBoNT-A and 20 U onaBoNT-A. (**B**) BoNT-A’s biological activity from baseline to 72 h of incubation with 50 U relaBoNT-A and 20 U onaBoNT-A. Results are presented for three individual batches of each drug product, measured at Galderma. BoNT-A, botulinum toxin serotype A; onaBoNT-A, onabotulinumtoxinA; relaBoNT-A, relabotulinumtoxinA; SNAP-25, synaptosome-associated protein 25.

When assessing on-label GL doses, relaBoNT-A (50 U) showed higher in vitro biological activity than onaBoNT-A (20 U) (11% versus 6% of cleaved SNAP-25 in motor neurons after 8.5 h of incubation) (Figure 5A). The in vitro assay results also showed earlier onset of SNAP-25 cleavage and consistently higher biological activity (percent of cleaved SNAP-25) with relaBoNT-A, compared with onaBoNT-A, for all incubation timepoints assessed up to 72 h (Figure 5B).

## 3. Discussion

This study demonstrated that the novel PEARL™ Technology produces a ready-to-use, liquid BoNT-A1 drug product, relaBoNT-A, which is characterized by high BoNT-A-specific potency (~2 × 10^8^ LD_50_ units/mg) that is consistently maintained throughout the drug substance manufacturing process, from initial bacterial culture through to the final purified drug substance. The consistently high specific potency achieved throughout the drug substance processing reflects the numerous and thorough purification steps during the manufacturing process, i.e., three filtration steps to remove cells, cell debris, and media components and four chromatography steps to remove nucleotides and unwanted host cell proteins (including complexing proteins) and purify the toxin (Figure 1). The PEARL™ manufacturing processes are rigorously controlled by In-Process Control (IPC) and release testing, including both microbiological methods such as microbial growth and endotoxin testing, as well as chemical and biochemical methods, including pH and conductivity control of critical buffer solutions. Identity and potency are just a few important tests of many that are performed on every produced batch of final drug substance, as well as drug product. Furthermore, two important parameters, the purity and specific potency, are specifically controlled in the release testing of the drug substance. The results presented in this paper showed consistent results for specific BoNT-A1 activity throughout drug manufacturing (Figure 2) and biological activity of the final product (Figure 5) for different batches of relaBoNT-A. Furthermore, as the relaBoNT-A product comes ready-to-use, which removes variability from the reconstitution step, this likely translates into vial–vial consistency for the final product.

The final relaBoNT-A drug product was shown to have high, at almost double the amount, BoNT-A proteolytic activity per on-label GL dose compared to onaBoNT-A (53 versus 29 BoTest^®^ units per GL dose). This is the result of the combination of a high amount of BoNT-A core protein and a high specific activity of the toxin molecules that are present in the final drug product: as shown, a standard GL dose of 50 U relaBoNT-A was determined to have 0.27 ng of BoNT-A core protein, compared to 0.18 ng for the on-label GL dose of 20 U of the commercial powder BoNT-A, onaBoNT-A. It was also shown that relaBoNT-A has higher specific BoNT-A proteolytic enzyme activity (BoTest units/mg of core neurotoxin protein), with onaBoNT-A displaying 81% of the specific activity of relaBoNT-A. We suggest that this combination of high core neurotoxin load together with high specific activity leads to a final high content of BoNT-A activity in the on-label GL dose of the relaBoNT-A product, which may explain the high efficacy rates and long duration of clinical effect observed in clinical trials, as described below. Finally, when assessing the on-label GL dose in a cell-based biological model, relaBoNT-A showed higher in vitro biological activity in cleaving SNAP-25 in motor neuron cells than onaBoNT-A (11% versus 6% of cleaved SNAP-25 after 8.5 h incubation), as well as consistently faster onset of effects across the triplicate batches of relaBoNT-A and onaBoNT-A that we assessed. Prior studies have reported amounts of BoNT-A core protein content per standard GL dose for other marketed products within the ranges reported here (aboBoNT-A, 0.27–0.32 ng; onaBoNT-A, 0.15–0.18 ng; incoBoNT-A, 0.08–0.09 ng) [34,35]. However, in the Field et al. publication [35], the specific activity of the BoNT-A core protein was found to not differ between previous marketed products tested. The increased specific activity of the BoNT-A core protein for relaBoNT-A relative to onaBoNT-A found in this study, together with the high amount of BoNT-A core protein, might suggest some differences between relaBoNT-A and prior products tested.

The in vitro characterization data presented here may help to explain the previously reported Phase 3 clinical data, which have shown that relaBoNT-A is well tolerated and highly effective at improving the esthetic appearance of facial lines. In the 6-month, randomized, double-blind, Phase 3 READY-1 trial involving 297 subjects with moderate-to-severe GLs, a single treatment of relaBoNT-A (50 U; *n* = 223) led to significant improvements in GL severity compared with placebo, with up to 39% of subjects seeing effects from Day 1 and up to 75% of subjects maintaining improvements for 6 months [30]. Similarly, in the 6-month, randomized, placebo-controlled, Phase 3 READY-2 trial involving 303 subjects with moderate-to-severe LCLs, relaBoNT-A (60 U; *n* = 230) treatment provided statistically significant improvement in LCL severity, with 34% of subjects reporting onset within one day and improvements being maintained through 6 months [32]. Moreover, in the randomized, double-blind, placebo-controlled, Phase 3 READY-3 trial, relaBoNT-A was well tolerated and demonstrated significant efficacy, long duration of effect, and high patient satisfaction versus placebo, as well as during treatment of GLs alone, LCLs alone, or GLs plus LCLs in combination [33]. The READY-4 Phase 3 study recently assessed the safety and efficacy of repeated injections of relaBoNT-A for the treatment of moderate-to-severe GLs and LCLs in 902 subjects. A comparable safety profile was observed with those seen in previous studies (READY-1–3) and other marketed BoNT-A products [36]. In addition, high efficacy and high levels of patient satisfaction were also maintained across repeated injections for up to four treatment cycles.

Overall, the in vitro and clinical data for relaBoNT-A reflect the benefits of PEARL^TM^ Technology and drug manufacturing, which was designed to maintain the core BoNT-A protein in a liquid form in its original conformation, minimizing any denaturation or unfolding and thus preserving its neuromodulator activity [27,28]. Furthermore, PEARL^TM^ Technology is designed to deliver a core neurotoxin (150 kDa) without any complex proteins. The ten-step PEARL^TM^ manufacturing process ensures a high-quality DS product by encompassing multiple filtration and chromatography steps, as well as a final bioburden filtration step to prevent microbial contamination. In a study examining the PEARL^TM^ process’s performance, an approximately 140-times-purer BoNT-A1 was obtained when comparing the drug substance to the harvested cultivation supernatant [27]. The final drug substance has been shown to be >98% pure in the main peak from ultra-high-performance liquid chromatography size exclusion chromatography, with SDS-PAGE results demonstrating that protein impurities diminish throughout the purification process, until no detectable impurities remain [27,28]. Preservation of the high-purity core toxin protein in the relaBoNT-A liquid formulation may explain the high specific enzyme activity, high biological activity, and rapid onset of effect seen in the in vitro settings. Different BoNT-A formulations have been shown to have non-equivalent activity and potencies [37,38,39]. In addition, within the clinical setting, BoNT-A products have shown dose-dependent effects on response rate and duration of effect [40], as well as subject satisfaction and psychological well-being [41], suggesting that the product potency may play a role in influencing the duration of the BoNT-A product [37,38,39].

Finally, most BoNT-A products that are commercially available are powders requiring reconstitution, which presents several potential challenges, including preparation time and issues with reconstitution, consistency, potential contamination, and precision of dosing [21,22,24,42]. Drug preparation errors related to reconstitution, such as wrong drug concentration, diluent, and inadequate aseptic technique, are an issue across many drug products that require reconstitution, with reported error rates for wrong concentrations ranging from 0.3 to 89% across studies [43,44]. Through the PEARL^TM^ Technology manufacturing process, relaBoNT-A is optimized for simple volumetric dosing, without reconstitution, to facilitate ease of use and ensure a consistent dose/volume of the product. Of note, in a study comparing the usability of a powder BoNT-A versus a liquid BoNT-A (not relaBoNT-A) in the treatment of GLs, clinical investigators reported that the liquid formulation reduced preparation time, made the injectors feel more precise in their injections, used less materials (which potentially has indirect environmental benefits due to saline bottles, reconstitution syringes, and needles needing to be disposed of with other botulinum toxin agents that are currently on the market), and allowed them more time to focus their attention on the patient [37]. The consistency of a liquid formulation could potentially be advantageous for clinics with larger patient volumes, reducing variability that could arise from being treated by multiple injectors. Further studies are needed to assess the relative usability and benefits of ready-to-use, liquid relaBoNT-A for both clinicians and patients.

This study was limited to an ELISA for quantifying the core neurotoxin content, an in vitro proteolytic BoNT-A enzymatic assay (BoTest), and a cell-based biological activity assay of relaBoNT-A for enzyme activity and speed of onset of effects. However, these in vitro characterization data may help explain the clinical efficacy and tolerability data seen in Phase 3 trials of relaBoNT-A for the treatment of GLs and LCLs [31,32,33,34,35,36]. Although the current in vitro data tend to suggest a potential for relaBoNT-A to provide faster onset of action and higher efficacy than onaBoNT-A (when comparing standard/on-label GL doses of each drug), caution must be taken in making comparisons; head-to-head, randomized, clinical trials are required to determine the clinical relevance of these in vitro data in clinical settings. Future studies linking the potency and clinical effects of relaBoNT-A—particularly in vivo studies which may allow for more standardized testing of this effect—could help clarify how these data are applicable to the clinical results seen in randomized Phase 3 trials.

## 4. Conclusions

In conclusion, the new PEARL^TM^ Technology preserves high specific enzyme potency throughout the drug substance manufacturing process and yields a high-quality complex protein-free product. The final novel, ready-to-use, liquid relaBoNT-A product has a higher amount of core protein per GL dose, in combination with higher specific endoprotease activity, leading to considerable higher SNAP-25 cleavage activity per dose and higher biological activity and faster onset of action in a cell-based assay than the powder onaBoNT-A product. Any clinical implications of potential differences between products need to be confirmed in a head-to-head clinical trial. However, these in vitro data indicate relaBoNT-A to be a high-quality product, combining a high load and a high specific activity of the core neurotoxin, which may help explain the efficacy and tolerability data previously reported in Phase 3, placebo-controlled, clinical trials of relaBoNT-A treatment of GLs and LCLs.

## 5. Methods

### 5.1. PEARL™ Technology

#### 5.1.1. Drug Substance Manufacturing Process

PEARL™ Technology comprises a ten-step process that aims to gently and efficiently manufacture a high-purity, complex-free/accessory protein-free, ready-to-use, liquid BoNT-A1 product. The process is carried out in a purpose-built manufacturing site at Galderma, Uppsala, Sweden, built to enable the highest possible level of quality control, with single-use technology and 100% renewable energy sources. Each step in the manufacturing process of the drug substance (DS) used in relaBoNT-A was designed for a specific purpose (Figure 1A). The first step is to cultivate a proprietary *Clostridium botulinum* type A1 strain. Next, three filtration steps (tangential flow filtration [TFF]) and four chromatography steps are performed to purify and separate the core neurotoxin from the bacterial proteins and from the neurotoxin complexing proteins. The third chromatographic step, which is an anionic exchange chromatography column, is run at a basic pH that allows for the separation of the neurotoxin molecule from the complexing proteins, including non-toxic non-hemagglutinating (NTNH) protein. The chromatography steps include ion exchange and size exclusion chromatography, with size exclusion chromatography as the final polishing step to remove truncated and aggregated versions to ensure that only core neurotoxin of native size is found in the final DS solution (Figure 1B).

#### 5.1.2. Drug Product Formulation

From the DS, a drug product (DP) bulk is obtained by precisely adding a calculated amount of DS to a carefully designed DP buffer containing sodium phosphate saline (sodium phosphate, KCl, NaCl) to achieve an isotonic and pH-stable solution and two important stabilizing additives: tryptophane and polysorbate 80. The DP bulk is sterile-filtered and then aseptically filled in glass vials to achieve a final formulated DP.

### 5.2. Measurement of Specific Potency of BoNT-A1 Throughout the PEARL^TM^ Drug Substance Processing

RelaBoNT-A was manufactured from the proprietary *Clostridium botulinum* type A1 strain using the PEARL^TM^ DS processing technology described above. To assess the specific potency of the BoNT-A1 (core protein) throughout the different steps of DS processing, samples were collected at four different points during the process (Figure 1B): one from the initial post-culture step, two at the early and mid-stages of DS production, and the fourth one from the final DS. From these four different sampling points, potency was determined using a standardized mouse LD_50_ assay, and toxin concentration was measured by an enzyme-linked immunosorbent assay (ELISA) for the unpure three first process fractions, while for the pure DS, the toxin concentration was measured by micro-bicinchoninic acid (µBCA, Thermo Scientific) per standard release testing. The specific potency (units/mg of toxin) was then calculated by dividing the potency value in units/mL by the toxin concentration in mg/mL. This was repeated for four batches from each process sampling point.

#### 5.2.1. BoNT-A (Core Protein) Amount in DS Process Fractions (ELISA)

A BoNT-A-specific ELISA was used to determine the concentration of BoNT-A in unpure DS process fractions. In the ELISA method, BoNT-A-specific rabbit antibodies coated in a microtiter plate are used to capture BoNT-A in the samples. Incubation with a BoNT-A-specific detection goat antibody is then followed by incubation with a horseradish peroxidase (HRP)-conjugated secondary rabbit antibody. The wells are washed between each step. A TMB (3,3′, 5,5′-tetramethylbenzidine) substrate is then added, which forms a blue color when oxidized by HRP. The color is measured spectrophotometrically at 450 nm. The BoNT-A concentration in the samples is determined by comparison to a standard curve on the plate, with known amounts of BoNT-A.

#### 5.2.2. Total Protein Concentration in Final DS (µBCA)

The total protein concentration was determined in accordance with the monograph in Ph. Eur. 07/2022: 2113 using the Micro BCA Protein Assay Kit from Thermo Scientific. Bicinchoninic acid (BCA) acts as a detection agent for Cu^1+^ that has been reduced from Cu^2+^ by the proteins in the test solution under alkaline conditions. One copper ion, Cu^1+^, and two molecules of BCA form a purple product, and absorbance is measured at 562 nm in a spectrophotometer. A standard curve from bovine serum albumin is used to calculate the concentration of protein from the measured absorbance. Note that no bovine or human serum albumin is part of the final formulation. The result is reported as the mean value of duplicate sample measurements. Due to background added from the DS buffer, a batch-specific DS buffer is used in the method to correct the absorbance values. For each sample, two separate dilutions are performed to be in the range of the standard curve, and each sample dilution is loaded in triplicate on the microtiter plate.

### 5.3. Characterization of Final relaBoNT-A Drug Product and Comparison with OnaBoNT-A

The final RelaBoNT-A drug product was characterized in vitro using three measures: (1) BoNT-A (core protein) amount measured using a commercially available sandwich ELISA (BoLISA; BioSentinel, Madison, WI, USA); (2) SNAP-25 cleavage using a commercially available fluorescence resonance energy transfer-based (FRET) enzyme activity assay, BoTest^®^ (BioSentinel, Madison, WI, USA) to measure enzyme activity isolated from other cellular mechanisms; (3) SNAP-25 cleavage using a neuronal cell-based assay, as described below, measuring the speed of internalization into the neuron as well as SNAP-25 cleavage activity. For each of these three measures, the results for relaBoNT-A were compared to similar measurements for commercial batches of the first approved powder BoNT-A product, onaBoNT-A. For the ELISA and BoTest^®^ enzyme activity assays, three batches of relaBoNT-A and three batches of onaBoNT-A were assessed. The batches for each product had a range of time to expiration of 0 to 15 months for relaBoNT-A and 6 to 26 months for onaBoNT-A. For each batch, two repeat measurements were performed independently at different independent laboratory sites (Uppsala University and Galderma).

#### 5.3.1. Sample Preparation for BoTest and BoLISA of Final Drug Products

To avoid any matrix effects due to different excipients in the two products, a final common buffer was used. As relaBoNT-A is a liquid formulation and onaBoNT-A is a powder, the sample preparation of each product was different. To match the buffer for both preparations, onaBoNT-A batches were reconstituted using relaBoNT-A DP buffer, and relaBoNT-A samples were prepared by mixing 360 µL of the ready-to-use DP with 40 µL of a solution containing 25 mg/mL human serum albumin and 45 mg/mL sodium chloride dissolved in DP buffer. For the BoTest assay, standard curve samples were prepared by diluting a stock solution of 421 mouse LD_50_ U/mL to eight different samples with a range of 34–200 mouse LD_50_ U/mL.

#### 5.3.2. BoNT-A (Core Protein) Amount in DP (BoLISA)

The quantity of BoNT-A core protein per on-label GL injection dose (50 U relaBoNT-A [29,32] and 20 U onaBoNT-A [11]) was measured using a sandwich ELISA with BoNT-A-specific antibodies (BoLISA; BioSentinel, Madison, WI, USA). Briefly, a BoNT-A-specific capture antibody (BioSentinel; 50 μL/well at concentration 4 μg/mL) was added to coat a 96-well microtiter plate and incubated overnight at 2–8 °C. Thereafter, triplicates of 100 μL/well of the relaBoNT-A and onaBoNT-A samples and BoNT-A standards were added to the wells and incubated for 70–90 min at room temperature. A biotinylated BoNT-A-specific detection antibody (BioSentinel; 100 μL/well at concentration 2.0 μg/mL) was then added and incubated for 60–70 min at room temperature. An HRP–streptavidin conjugate (Fisher Scientific; 100 μL/well) was then added and incubated for 60 min at room temperature. The conjugate will interact with the biotinylated antibody, where the HRP will convert the substrate used for detection. The wells were washed 6 or 12 times between each step. Next, a 3.3′, 5.5′-tetramethylbenzidine substrate (Merck; 100 μL/well) was added and incubated for 10 min, generating a blue color, the color intensity of which is proportional to the amount of HRP and thereby to the amount of BoNT-A in the sample. The reaction was stopped by adding a stop solution containing sulfuric acid (Fisher Scientific), which caused a color change to yellow in the samples. The color intensity was read at 450 nm using a 96-well plate reader. Finally, based on the standard curve, the relative BoNT-A1 concentration of the sample was calculated in pg/mL.

#### 5.3.3. BoNT-A Enzyme Activity in Final Product (BoTest)

The enzyme (endoprotease) activity of the core protein light chain of relaBoNT-A and onaBoNT-A was measured using a FRET protease assay, BoTest^®^, which quantifies BoNT types A and E. To enable a comparison between drug products, the enzyme activity results were normalized by (1) the on-label GL injection dose for each product (50 U relaBoNT-A [29,32] and 20 U onaBoNT-A [11]); (2) the amount of BoNT-A core protein in pg measured by ELISA (specific enzyme activity).

For the BoTest assay, 40 µL of standard samples and relaBoNT-A and onaBoNT-A samples were added to a 1 mL 96-well microtiter plate before adding 360 µL of the reaction mixture (1x BoTest^®^ reaction buffer, 5 mM dithiothreitol, 0.25 µM BoTest^®^ A/E reporter). After mixing, 100 µL was transferred in triplicates to a black flat-bottom 96-well microtiter plate, followed by a 16–20 h incubation at room temperature covered in aluminum foil. The analysis was performed using fluorescence with excitation at 434 nm and detection of emission at both 470 nm and 526 nm on all standard samples and test samples. A result for each sample was obtained by dividing the signal at 526 nm with the signal at 470 nm. A linear regression analysis was performed on the standard samples using the result from the analysis and the concentration in LD_50_ U/mL. The equation given from the linear regression was used to determine the concentration of the samples analyzed.

#### 5.3.4. BoNT-A’s Biological Activity in Final Product (Cell-Based Assay)

BoNT-A’s biological activity (binding, internalization, and SNAP-25 cleavage within cells) was measured using a cell-based assay. Human motor neurons from Fujifilm Cellular Dynamics (fCDI) were cultivated for 7 days and subsequently incubated with on-label doses of relaBoNT-A or onaBoNT-A drug product for up to 72 h, using a common buffer solution. The motor neurons were all from the same healthy donor, which is kept as a clone by fCDI and supplied by fCDI as motor neuron precursors together with differentiation and maturation media. Protein samples, using a radioimmunoprecipitation assay buffer supplemented with protease and phosphatase inhibitors, were made from the cells. The amount of cleaved SNAP-25 in the cell samples was assessed with a Western blot method using an anti-SNAP-25 antibody that is capable of detecting both whole and cleaved SNAP-25. The imaging was performed using chemiluminescent detection in a CCD camera (Chemidoc XRS+ Imaging System, Biorad). On-label GL injection doses of each drug (50 U relaBoNT-A [29,32] and 20 U onaBoNT-A [11]) were used for the incubations to allow for comparison between the BoNT-A products. The data are presented as percent of cleaved SNAP-25.

#### 5.3.5. Statistics

Statistical calculations were made using SAS^®^ analysis software, version 9.4 (SAS Institute Inc., Cary, NC, USA). Differences between drug products were compared for statistical significance by using a repeated-measures models with fixed effects for the treatment group and laboratory, and batch as the repeated factor. A significance level of 0.01 was used to account for the small sample size.

## Figures and Tables

**Table 1:** Data on BoNT-A1 product batches tested at different sites.

BoNT-A Product	Batch	BoNT-A1 Concentration (pg/mL) ^(1)^	Enzyme Activity (BU/mL) ^(2)^	Specific Enzyme Activity ^(3)^ (BU/pg BoNT-A)	Enzyme Activity (BU)/GL Dose ^(3)^	BoNT-A Amount (pg)/GL Dose ^(3)^
Measurements by Galderma						
RelaBoNT-A ^(4)^	19394	497	108	0.218	54	249
	20868	488	108	0.220	54	244
	21457	572	101	0.177	50.5	286
	Mean (SD)	519 (46)	106 (3.9)	0.205 (0.02)	52.8 (1.9)	260 (23)
OnaBoNT-A ^(4)^	C7223C2	761	138	0.181	28	152
	C6463C2	919	163	0.177	33	184
	C7932C2	862	143	0.166	29	172
	Mean (SD)	847 (80)	148 (13)	0.175 (0.01)	29.6 (2.7)	169 (16)
Measurements by Uppsala University						
RelaBoNT-A ^(4)^	19394	549	103	0.188	52	275
	20868	548	96	0.176	48	274
	21457	572	119	0.207	59	286
	Mean (SD)	556 (14)	106 (11)	0.190 (0.02)	53.0 (5.7)	278 (6.9)
OnaBoNT-A ^(4)^	C7223C2	910	128	0.141	26	182
	C6463C2	1069	162	0.151	32	214
	C7932C2	862	124	0.143	25	172
	Mean (SD)	947 (108)	138 (21)	0.145 (0.01)	27.6 (4.2)	189 (22)
Combined Measurements from Galderma and Uppsala University ^(5)^						
RelaBoNT-A ^(4)^	LSMean (99% CI)	538 (446, 630)	106 (88, 124)	0.198 (0.178, 0.218)	52.9 (48.0, 57.9)	269 (246, 292)
OnaBoNT-A ^(4)^	LSMean (99% CI)	897 (805, 990)	143 (125, 161)	0.160 (0.140, 0.180)	28.6 (23.6, 33.5)	179 (157, 202)
Difference (RelaBoNT-A ^(4)^ − OnaBoNT-A ^(4)^)	LSMean (99% CI)	−359 (−490, −228)	−37.0 (−62.0, −11.9)	0.038 (0.009, 0.066)	24.4 (17.3, 31.4)	89.5 (57.6, 121.5)
	Effect Size	−5.16	−2.77	2.50	6.52	5.26
	*p*-value	<0.0001	0.0010	0.0019	<0.0001	<0.0001

BU, BoTest units; GL, Glabellar line; LS, Least squares. ^(1)^ BoNT-A1 concentration was measured by BoLISA; ^(2)^ Enzyme activity = endoprotease activity as measured by BoTest; ^(3)^ calculated values. ^(4)^ The values for onaBoNT-A correspond to the product reconstituted to 100 U/mL and for ready-to-use relaBoNT-A liquid as delivered by the manufacturer. ^(5)^ Results based on repeated-measures models to account for the dependence of the data from Galderma and Uppsala University.

## Data Availability

The original contributions presented in this study are included in the article. Further inquiries can be directed to the corresponding authors.

## Abbreviations

aboBoNT-A: abobotulinumtoxinA; BCA, bicinchoninic acid; BoNT-A, botulinum neurotoxin serotype A; BoNT-A1, BoNT-A subtype 1; CI, confidence interval; DP, drug product; DS, drug substance; ELISA, enzyme-linked immunosorbent assay; ES, effect size; EU, European Union; FRET, fluorescence resonance energy transfer; GLs, glabellar lines; HRP, horseradish peroxidase; IEX, ion exchange chromatography; incoBoNT-A, incobotulinumtoxinA; LCLs, lateral canthal lines; LD_50_, median lethal dose; LS, least squares; onaboNT-A, onabotulinumtoxinA; PEARL™, Precipitation-free Extraction and Activity-preserving, Refined Liquid; praBoNT-A, prabotulinumtoxinA; relaBoNT-A, relabotulinumtoxinA; SEC, size exclusion chromatography; SNAP-25, synaptosome-associated protein-25; SNARE, soluble N-ethylmaleimide-sensitive factor attachment receptor; TMB, tetramethylbenzidine; TFF, tangential flow filtration; U, units.
